# Tools for regulating metabolic diseases: extracellular vesicles from adipose macrophages

**DOI:** 10.3389/fendo.2024.1510712

**Published:** 2024-12-10

**Authors:** Liang Zhang, Kecheng Lou, Yunmeng Zhang, Yuanjing Leng, Yuqing Huang, Xinxin Liao, Xiaoliang Liu, Shangzhi Feng, Guoqiang Feng

**Affiliations:** ^1^ Department of Urology, Jiujiang University Clinic College/Hospital, Jiujiang, Jiangxi, China; ^2^ Department of Urology, Lanxi People’s Hospital, Jinhua, Zhejiang, China; ^3^ Department of Anesthesiology, Jiujiang College Hospital, Jiujiang, Jiangxi, China; ^4^ Department of Rehabilitation, Jiujiang College Hospital, Jiujiang, Jiangxi, China

**Keywords:** obesity, metabolic disease, adipose tissue macrophage, extracellular vesicles, nano-targeted therapy

## Abstract

Metabolic diseases have gradually become one of the most significant global medical burdens. Diseases such as obesity, diabetes, and metabolic syndrome, along with their complications, are clinically categorized as metabolic diseases. Long-term oral medication significantly reduces patient compliance and quality of life. Therefore, alternative therapies that intervene at the cellular level or target the root causes of metabolic diseases might help change this predicament. Research has found that extracellular vesicles derived from adipose macrophages can effectively regulate metabolic diseases by influencing the disease’s development. This regulation is likely related to the role of these extracellular vesicles as important mediators in modulating adipose tissue function and insulin sensitivity, and their involvement in the crosstalk between adipocytes and macrophages. This review aims to describe the regulation of metabolic diseases mediated by adipose macrophage-derived extracellular vesicles, with a focus on their involvement in adipocyte crosstalk, the regulation of metabolism-related autoimmunity, and their potential as therapeutic agents for metabolic diseases, providing new avenues for diagnosis and treatment.

## Introduction

Non-communicable chronic metabolic diseases have increasingly become major global public health issues, imposing a significant burden on healthcare worldwide (1). Current understanding suggests that a series of processes affecting metabolic imbalance are metabolic diseases. These include conditions like hypertension, type 2 diabetes, hyperlipidemia, obesity, non-alcoholic fatty liver disease, and their related complications (2). These diseases often coexist and share numerous common risk factors, ultimately leading to irreversible outcomes such as disability, death, and an increased risk of cancer (3, 4). Global metabolic statistics indicate that obesity represents the largest burden among metabolic diseases, with its prevalence steadily increasing over the past two decades. The accumulation of fat caused by obesity has become a significant factor contributing to metabolic disorders and related diseases (1). In fact, obese individuals are considered to have a higher risk of death, including mortality associated with obesity-induced cardiovascular diseases, diabetes, inflammatory conditions, and their complications (5). In terms of insulin resistance, the accumulation of pro-inflammatory macrophages in adipose tissue is a crucial factor leading to obesity-associated insulin resistance (6). Moreover, the systemic low-grade chronic inflammation seen in obese individuals is a potential consequence that causes long-term chronic damage to multiple metabolic organs (7).

With the rising global obesity rates, obesity-induced metabolic diseases increasingly impact people’s quality of life. Understanding how fat mediates the development of metabolic diseases has thus become an interesting area of research. In reality, changes in cell types within adipose tissue (AT) (such as inflammatory cells and vascular endothelial cells) and variations in cytokines (like leptin and miRNA) are closely related to the development of fat-induced metabolic diseases. Macrophages in AT are likely key players in promoting adipose inflammation and may also be important mediators of the crosstalk between AT and metabolic diseases. Extracellular vesicles (EVs) are considered critical tools in transmitting cytokines that promote obesity-related metabolic diseases. For example, recent discoveries suggest that AT macrophages regulate metabolic and inflammatory interactions between adipocytes and distal tissues via a novel mechanism involving the secretion of EVs (8). Therefore, adipocyte-macrophage extracellular vesicles may have potential functions in regulating fat and metabolic diseases, presenting a new avenue for treating obesity-related metabolic diseases.

## EVs mediate the occurrence and development of metabolic diseases

The regulation of systemic metabolic processes results from the interactions between key metabolic tissues, including AT, the liver, and skeletal muscle. Metabolic dysfunction includes a variety of disease risk factors that significantly increase the risk of cardiovascular diseases such as acute myocardial infarction and stroke. The comprehensive pathogenesis of metabolic dysfunction involves multiple cell types, tissues, organs, inflammatory signaling cascades, and humoral factors. Research indicates that metabolic dysfunction is related to changes in plasma EV concentrations and their cargo. EVs produced by cells in metabolic tissues can potentially carry all biomolecules involved in the mechanisms of metabolic dysfunction, ultimately promoting the onset of metabolic diseases. EVs can also act as messengers between donor and recipient cells, potentially participating in communication between tissue cells and organs during metabolic diseases. This suggests a close association between the occurrence and development of metabolic diseases and changes in EVs. In fact, several studies have found that EVs have great potential value as biomarkers for prognosis and diagnosis in metabolic diseases (**Table 1**). Moreover, because EVs can carry mRNA and microRNA (miRNA) to modify the gene expression of recipient cells, EVs might offer a means of repairing damaged metabolic tissue cells at the genetic level, sparking great interest in the role of EVs in improving metabolic disorders.

**Table 1:** EVs regulate the development of metabolic diseases.

Type of Disease	Species	EV Source	Role	Ref.
Diabetic nephropathy	Human	Urine	Involved in diabetic nephropathy	(9)
Gestational Diabetes	Human	Plasma	Involved in placental connections to various maternal organs/cells	(10)
Type 2 Diabetes	Mice	Macrophages from adipose tissue	Involved in the regulation of insulin sensitivity	(8)
Type 2 Diabetes	Mice	Brown adipocytes	Involved in glucose metabolism injury	(11)
Type 2 Diabetes	Human	Pancreatic islets	Involved in monitoring pancreatic islet function	(12)
Type 2 Diabetes	Human	Skeletal muscle	Inhibited progression of insulin resistance	(13)
Type 2 Diabetes	Mice	Visceral adipose tissue	Involved in insulin resistance and tissue inflammation	(14)
Type 2 Diabetes	Rat	Cardiac muscle cells	Involved in anti-angiogenesis	(15)
Hepatocellular carcinoma	Human	HCV-infected liver	Involved in lipid metabolism	(16)
Hypothyroidism	Human	Endothelial cells	Involved in thyroid impairment	(17)
Nonalcoholic Fatty Liver Disease	Human	Damaged hepatocytes	Involved in endothelial angiogenesis	(18)
Type 1 Diabetes	Human	Plasma	Involved in type 1 diabetes development	(19)
Hepatocellular carcinoma	Human	Serum	Manipulated Hepatocellular Carcinoma stemness and invasiveness	(20)
Obesity	Mice	Adipose Stem Cells	Involved in M2 macrophage polarization	(21)
Obesity Related Liver Disease	Human	Adipocytes	Involved in TGF-β signaling pathway dysregulation	(22)
Post-surgical diabetes mellitus	Human	Adipocytes from blood	Involved in postoperative insulin resistance	(23)
Type 2 Diabetes	Mice	Skeletal muscle	Involved in β-cell proliferation during insulin resistance	(24)
Fatty Liver Disease	Human	Liver tissue	Involved in hepatocyte death, fibrosis and pathological angiogenesis	(25)
Hypertension	Rat	Serum	Involved in induction of hypertensive-type endothelial cell inflammation	(26)

## EVs regulate glucose metabolism

Globally, nearly 400 million people have type 2 diabetes (T2DM) (7, 27), T2DM is considered a multifactorial disease, with its onset related to genetic factors and lifestyle choices, such as high-fat intake, alcohol consumption, and smoking, which lead to obesity (28). The prevalence of T2DM rises in tandem with obesity, with meta-analysis results from the United States and Europe showing that obese men and women are seven and twelve times more likely to develop T2DM than their lean counterparts, respectively. This indicates a strong correlation between obesity and T2DM (29). However, the molecular mechanisms underlying the link between obesity and T2DM are not fully understood. Studies have found that the pathogenesis of T2DM is closely related to dysfunctions in adipose tissue macrophages (ATMs), particularly alterations in macrophage metabolism that lead to AT inflammation and obesity (6). The chronic systemic inflammation associated with obesity is an important cause of insulin resistance and the onset of type 2 diabetes mellitus (T2DM) (28). This ongoing low-grade inflammation is thought to contribute to changes in insulin-glucose homeostasis related to obesity. An important observation is that in obese mice and humans, increased levels of inflammatory cytokines (such as tumor necrosis factor-α and interleukin-6) in AT have been found to lead to insulin resistance (30). Many obese individuals are in a pre-diabetic state, eventually progressing to T2DM characterized by insufficient insulin secretion. In obesity, AT undergoes significant expansion, accompanied by a chronic and unresolved inflammatory state (31). Furthermore, a significant cause of tissue inflammation response induced by obesity is the accumulation of pro-inflammatory macrophages, particularly in AT and the liver (8). Numerous studies in humans and rodents have shown that significant accumulation of pro-inflammatory macrophages is a major component of the AT inflammation response induced by obesity (31). These pro-inflammatory macrophages present in obese adipose tissue are major drivers of the pathogenesis of tissue inflammation and insulin resistance induced by obesity (31). It was further found that chronic tissue inflammation caused by the accumulation of M1 macrophages is a critical marker of insulin resistance, and the influx of pro-inflammatory M1 macrophages into AT is an important contributor to AT and obesity-associated insulin resistance (30). For example, M1 macrophages and insulin-resident macrophages in obese mice secrete EVs enriched with miR-212-5p, which can impair insulin secretion by β cells (32).

AT is considered a major source of circulating EVs miRNA (33). EVs-mediated cellular communication plays a profound regulatory role in the metabolic response to obesity (34, 35). Studies have shown that obese ATMs can decrease peripheral insulin sensitivity by releasing EV/microRNA (miRNA) either locally or into circulation (31). The risk of developing type 2 diabetes is associated with adipocyte hypertrophy (36) and increased production and release of adipocyte EVs, characterized by changes in the expression of perilipin A (37, 38). Consistent with these studies, circulating levels of adipocyte-derived EVs are increased in obese mice and humans and decrease following energy restriction or weight loss surgery (23). Interestingly, 55 types of adipocyte-derived EVs miRNAs have been identified that are differentially expressed between obese and lean individuals, suggesting that in addition to their higher circulating levels, the cargo of EVs is also regulated in obese individuals (23). Furthermore, obesity and insulin resistance are also associated with the accumulation of macrophages in AT (39). A large body of literature describes how ATMs play a detrimental role in regulating systemic metabolism by overproducing inflammatory cytokines that can block insulin signaling (40).

Obesity is a major risk factor for insulin resistance, which promotes the development of T2DM. Obesity-associated insulin resistance is a precursor to type 2 diabetes (27). This may be due to the close correlation between the number of resident ATMs and the degree of insulin resistance and metabolic disturbances. For example, selective depletion of ATMs through genetic or pharmacological methods can significantly prevent obesity-associated insulin resistance and metabolic complications in obese mice (41). Interestingly, EVs released by ATMs are also involved. Studies have found that administration of miRNA-containing EVs secreted by ATMs from obese mice to lean mice causes glucose intolerance and insulin resistance. Conversely, administration of ATMs EVs obtained from lean mice to obese mice can improve glucose tolerance and insulin sensitivity. Specifically, the miRNAs in these EVs can be transferred to insulin target cell types through paracrine or endocrine regulatory mechanisms, having a strong impact on cellular insulin action, *in vivo* insulin sensitivity, and overall glucose homeostasis (8). For instance, ATM-EVs containing miRNAs can regulate systemic insulin and glucose tolerance by directly affecting cellular insulin signaling. Thus, when lean insulin-sensitive mice are treated with obese ATMs-EVs, they develop systemic insulin resistance and glucose intolerance. In contrast, treatment with lean ATMs-EVs in obese insulin-resistant mice can lead to near-normalization of glucose tolerance and improvement of systemic insulin sensitivity (8). Similar results have been found *in vitro*, where EVs released by healthy 3T3-L1 adipocytes can enhance the survival and proliferation of INS-1E β-cells and human islets by stimulating insulin secretion. In contrast, EVs derived from inflamed adipocytes carrying low levels of miR-296-3p, miR-298-5p, miR-351-5p, and miR-125a-5p lead to β-cell death and dysfunction, while EVs rich in miR-155-5p from obese human AT (including ATMs) result in β-cell death and dysfunction (23).

## EVs regulate lipid metabolism

Dysregulation of specific circulating EVs miRNAs involved in lipid metabolism regulation pathways may exist in patients with metabolic syndrome or individual metabolic diseases. The characteristics of EVs released from 3T3-L1 cells during adipocyte differentiation show stage-specific changes in lipid and protein content, as well as in the number and size distribution of EVs during differentiation (42). This suggests that the signaling functions of pre-adipocytes and mature adipocytes differ in adipocyte EVs. One signaling function of adipocyte-EVs might be to communicate with other cells in adipose tissue (including fibroblasts, pre-adipocytes, endothelial cells, and immune cells) to coordinate the response of tissue cells to different fuel availabilities, such as lipid metabolism (43). During obesity, AT dysfunction results from adipocyte stress, characterized by hypertrophy and hypoxia. One study found that hypoxia affects the composition of adipocyte EVs cargo by increasing levels of proteins related to metabolic processes, particularly enzymes associated with *de novo* lipogenesis; these EVs were found to increase lipid accumulation in recipient adipocytes (44). Similarly, other studies have shown that microvesicles containing CD73 released by adipocytes *in vitro* can stimulate lipid synthesis in recipient small adipocytes (45). The transfer of adipogenic mechanisms may represent a burden-shifting of lipid storage from hypertrophic adipocytes to recipient and non-stressed adipocytes. Although no evidence was found for the transfer of insulin resistance between muscle cells via EVs, the myotube phenotype and myoblast proliferation in obese individuals were affected. This suggests that the adverse consequences of lipid-rich diets in obese individuals can be transmitted between skeletal muscle cells via EVs, leading to systemic cellular metabolic disturbances (46).

White adipose tissue (WAT) dysfunction is considered a major driver of obesity-related metabolic diseases (47). Changes in the contents of extracellular vesicles released by WAT can indicate the onset of metabolic diseases. By analyzing the RNA and protein content of WAT-derived EVs, the expression of adipocyte-specific and adipocyte-dominant proteins, such as fatty acid-binding protein 4 (FABP4) and adiponectin, can be found (42, 48, 49). The expression of some adipocyte markers, such as adiponectin associated with microvesicles, constantly changes during adipogenesis and differentiation (42), which is likely related to intercellular signaling communication involving EVs. For instance, removing EVs containing CD73 from adipocyte culture media can eliminate the pro-lipogenic effects of adipogenic stimuli (50). These EVs contain, in a dose-dependent manner, transcripts and miRNA involved in the up-regulation of adipogenesis (e.g., diacylglycerol acyltransferase-2) and lipid droplet assembly (e.g., caveolin-1 and perilipin-A) (50). Interestingly, when applied to cultured adipocytes, the effects on small adipocytes are greater than those on large adipocytes (50). This might result from the regulation of the nutritional and lipid-filled status of adipocytes being transmitted to their neighboring cells via EVs.

Compared to lean mice, the number of lipid-filled EVs secreted by adipocytes in obese mice more than doubled, which could be another mechanism of obesity-associated adipose inflammation. These lipid-filled EVs represent a novel pathway for adipocyte lipid release and are not dependent on typical lipolysis. However, little is known about how different types of bioactive lipids selectively enrich in adipocyte EVs and exert their local and/or distal effects on immune and metabolic regulation (41). Recently, a novel mechanism pathway involving extracellular vesicles secreted by ATMs has been found to regulate metabolic and inflammatory interactions between adipocytes, macrophages, and distal tissues. Notably, during obesity, ATMs undergoes significant changes in number, location, and inflammatory state (43). When incubated with EVs from human adipocyte lines and AT explants, monocytes differentiate into ATMs-like macrophages, and the conditioned media from these macrophages can inhibit adipocyte insulin signaling *in vitro* experiments (51). Regarding lipid metabolism, adipocyte-secreted EVs loaded with lipids can express lipid droplet-associated proteins such as perilipin1, phospholipids, neutral lipids, and free cholesterol; these lipids are taken up by ATMs and can induce bone marrow-derived precursor cells to differentiate into ATMs (30). Pro-inflammatory pathways in ATMs may impair glucose tolerance in obese patients, but ATMs may also serve as a reservoir for excess lipids that adipocytes cannot store. For example, the inability of obese individuals to appropriately expand their AT reservoir may lead to ectopic lipid deposition in the liver and skeletal muscle, which could be one of the causes of insulin resistance (52).

## EVs improve metabolic disease complications

With the rapid development of society and economy, due to overnutrition and lack of exercise, obesity has become a serious public health issue. Obesity is associated with various chronic diseases and significantly affects patients’ life expectancy (53). Cardiac remodeling and dysfunction caused by obesity without coronary heart disease and hypertension complications are referred to as obesity-related cardiomyopathy and are considered to lead to sudden cardiac death (54, 55). Studies have found that ATMs-EVs in obese individuals may be involved in the occurrence of complications following metabolic disorders. For example, ATMs-EVs can induce abnormal left ventricular systolic function in obese mice. It has been found that miR-140-5p is abundant in ATMs-EVs of obese individuals, which can promote ferroptosis in cardiomyocytes. Specifically, it induces ferroptosis by targeting SLC7A11 to inhibit GSH synthesis. Reducing the expression of miR-140-5p in ATMs-EVs can alleviate obesity and prevent ferroptosis and heart damage by mitigating GSH inhibition (56). Notably, peripheral ATMs-EVs can trigger microglia autophagy by inhibiting the PI3K/AKT/mTOR signaling pathway, promote anti-inflammatory microglial polarization, and stimulate anti-inflammatory properties, showing great potential for post-injury repair in metabolic diseases (57). However, due to insufficient targeting capability, the clinical application of unmodified peripheral ATMs-EVs is limited (58).

The occurrence of complications in metabolic diseases is a major reason for the strict control of metabolic disease progression. Obesity is often associated with low-grade inflammation, which determines the appearance of complications such as atherosclerosis and insulin resistance. ATMs from healthy lean donors have been found to improve glucose tolerance and insulin sensitivity and regulate related metabolic complications. In T2DM patients, M1 macrophages predominate, leading to excessive and prolonged inflammation at wound sites (59). This is due to the accumulation of M1 macrophages creating a harmful microenvironment, continuously promoting proteolysis and cellular damage (60). Therefore, regulating macrophage polarization will help in the healing of diabetic wounds. There are differences in miRNA expression in AT between obese and lean donors, and the levels of different miRNAs correlate with BMI to varying degrees (61, 62). EVs isolated from relatively lean donor AT drive macrophage polarization towards the M2 phenotype, resulting in relatively reduced inflammation (21). Macrophages are the most important immunomodulatory cells involved in the four phases of wound healing (hemostasis, inflammation, proliferation/repair, and remodeling) (60). Diabetic wounds continuously exhibit dysfunctional and M1 (pro-inflammatory) macrophage polarization, whereas normal wounds show a transition to M2 (pro-healing) macrophages (63). For instance, ATMs-EVs isolated from AT-conditioned media not only lead to the accumulation of miR-222-3p in macrophages but also induce the conversion of M1 macrophages to M2 macrophages by activating transcriptional programs with M2 phenotypic characteristics, thereby improving wound healing. *In vivo* experiments also show similar results, where ATMs isolated from lean mice can also secrete miRNA-containing EVs. When given to diabetes-prone mice, they regulate macrophage polarization and promote rapid healing of diabetic wounds. This suggests that changes in ATMs-EVs expression can lead to macrophage repolarization of diabetic wounds, providing new targets for promoting the healing of chronic diabetic foot (63). Additionally, during the proliferation phase, macrophage phenotype conversion inhibits inflammation while promoting angiogenesis (64), and ATMs-EVs can also promote the healing of diabetic wounds by accelerating angiogenesis and epithelialization processes. However, the mere quantity of angiogenesis is not sufficient to counteract the defects caused by macrophage polarization. Therefore, this may not be the primary factor in diabetic wound formation (65).

## ATM-EVs mediate adipose-macrophage crosstalk

Macrophages are inherently highly plastic, exhibiting different phenotypes in response to environmental changes, ranging from classically activated pro-inflammatory M1 to selectively activated anti-inflammatory M2 (66). Obesity leads to changes in the internal environment, which is one of the factors causing macrophage phenotype changes. Obesity induces significant phenotypic changes in ATMs, shifting from anti-inflammatory M2 to pro-inflammatory M1, which produces pro-inflammatory cytokines, exacerbating the occurrence and progression of metabolic diseases (66). In terms of obesity, there are reports of differences in miRNAs contained in EVs released from AT in control, leptin-deficient obese, and high-fat-fed obese mice (67). These exosomes secreted into the medium seem to have local and systemic effects and are absorbed by ATMs, enhancing their activation in AT. Due to this activation, more macrophages can be recruited to the AT and feedback the inflammatory response. Various vesicles released by adipocytes are likely key mediators, whose vesicle components mediate the polarization and immune regulatory response of resident ATMs in a paracrine manner (41). For example, EVs released from human adipocyte cultures can induce monocyte differentiation into ATMs-like macrophages *in vitro*, and adiponectin-positive EVs from human AT are more effective in promoting monocyte differentiation into ATMs than adiponectin-negative ones. This is because adiponectin-positive EVs are more capable of inducing monocyte differentiation *in vitro* and exhibit characteristics of ATMs (51). Additionally, EVs derived from adipocytes isolated from high-fat diet (HFD)-fed mice can drive the polarization of macrophages towards a pro-inflammatory M1 phenotype in bone marrow-derived macrophages (BMDM) *in vitro* through miR-155, thereby inhibiting the suppressor of cytokine signaling 1 (SOCS1), which in turn leads to inhibition of signal transducer and activator of transcription 6 (STAT6) (68). Moreover, adipocytes also mediate the growth of adjacent adipocytes through EVs, with adipocytes delivering EVs proteins to nearby preadipocytes and adipocytes in a paracrine and autocrine manner to regulate adipogenesis. Adipose-derived stem cells (ADSC) impart paracrine effects mediated by EVs on adipocytes and ATMs, respectively regulating adipocyte reprogramming and macrophage polarization. Indeed, the content of EVs may serve as a mediator of paracrine crosstalk between adipocytes and macrophages in AT. Studies have found that adipocytes release lipid-filled EVs, and these lipid-rich EVs play a significant role in transporting lipids from adipocytes to macrophages (34). Moreover, these lipid-filled EVs are sufficient to induce bone marrow-derived monocytes to differentiate into ATMs-like macrophages *in vitro* (34).

In fact, macrophages also potentially influence the structure of AT through EVs, leading to the occurrence of metabolic diseases. Macrophages can be abundantly stored in AT and interfere with adjacent adipocytes through EVs. Macrophage-derived EVs can effectively internalize into adipocytes, which may be a primary factor in the chronic inflammatory structure of AT (41). In *in vitro* experiments, EVs secretion can be detected in the medium of human THP-1-derived macrophages, and when applied to adipocyte culture dishes, internalization of EVs into adipocytes can be clearly observed through fluorescence labeling (69, 70). Similarly, a large number of EVs have also been isolated from the AT of obese mice and have been shown to be gradually absorbed by adipocytes (8). This is further evidenced by detecting EVs membrane markers, including TSG101, syntenin 1, CD63, and CD9. Interestingly, when THP-1 monocyte-derived macrophages are polarized into M1 or M2 phenotypes by LPS plus IFN-γ or IL-4, respectively, EVs derived from M1 macrophages impair insulin signaling in human adipocytes, while EVs derived from M2 macrophages enhance insulin signaling and glucose uptake in adipocytes (69). Consistent with *in vitro* study results, treatment with ATMs-derived EVs from lean mice can improve diet-induced glucose intolerance and insulin resistance in obese mice, whereas EVs isolated from ATMs of obese mice can promote glucose intolerance and insulin resistance in obese mice (8). However, EVs secreted by native macrophages do not affect the differentiation process of adipocyte precursor cells into adipocytes, fat storage, or insulin-mediated glucose uptake in adipocytes. This may be due to macrophage phenotype changes in the obese state. Indeed, changes in adipocyte gene expression induced by macrophage EVs depend on their origin (LPS-activated or non-activated macrophages), where lean AT is dominated by M2 macrophages, which maintain tissue homeostasis by phagocytizing dead adipocytes, secreting anti-inflammatory cytokines, and other angiogenesis, adipogenesis, and adaptive thermoregulation factors (71). In contrast, obese AT is dominated by M1 macrophages, causing metabolic disorders in body tissues. In summary, these studies collectively support the critical role of ATMs-derived EVs in regulating adjacent adipocytes under physiological and pathological conditions (41).

However, the exact process by which cells communicate through these vesicles has not been characterized, and the exosomal crosstalk pathway between macrophages and adipocytes remains unknown (70). While changes in adipocyte gene expression have been observed, it will also be necessary to identify specific molecules (i.e., mRNA and proteins) present in macrophage-derived EVs that affect adipocyte gene expression and physiological characteristics. Of course, if similar results are obtained in human AT, other macrophages, and adipocyte primary cell models, it would also be an interesting finding (70).

## ATM-EVs mediate crosstalk in adipose and metabolic diseases

Metabolic dysfunction is associated with AT inflammation and macrophage infiltration, ultimately leading to systemic metabolic dysregulation. New evidence suggests that obesity is accompanied by macrophage infiltration in AT, leading to low-grade chronic inflammation and a state of metabolic dysregulation. Recently, it has been proposed that adipocyte-derived EVs are involved in adipocyte/macrophage crosstalk and act as significant mediators in regulating the polarization of ATMs in obesity through adipocytes (72). It has been observed through protein fluorescence tracing that the intake of melatonin increases the content of α-ketoglutarate (αKG) in adipocyte-derived EVs, which is subsequently transported to macrophages, promoting the activation of M2 macrophages (73). In obesity, the phenotype of AT macrophages shifts from an M2 polarized state to an M1 state, leading to chronic inflammation and ultimately causing metabolic disorders. It has been found that ATMs undergo significant changes in number, location, and inflammatory status during obesity (43). When incubated with EVs from human adipocyte lines and adipocyte EVs, monocytes differentiate into ATMs-like macrophages, and conditioned media from these macrophages inhibit insulin signaling in adipocytes *in vitro* (51). Consistently, EVs from M1-like pro-inflammatory macrophages have been found to reduce insulin signaling in human adipocytes, potentially mediated by nuclear factor kappa B (NF-κB) activation, while M2-like derived EVs have the opposite effect (69).

Under physiological conditions, the body maintains metabolic homeostasis partly through communication between metabolic organs. Typically, this crosstalk is mediated by hormones or metabolites, but recently this has extended to EVs. Under physiological and pathological conditions, EVs participate in inter-organ communication by encapsulating a variety of biologically active substances. The processes of biogenesis, secretion, and specific cargo sorting of EVs are strongly influenced by dynamic physiological and pathological conditions. For example, a cell’s glucose metabolism status highly influences EVs secretion by mediating the sorting of cargo proteins into vesicles (74). Certain endocrine and metabolic factors, such as hydrocortisone, insulin, and cholesterol, can also affect the secretion or cargo composition of EVs. Hydrocortisone, as a corticosteroid, can enhance the secretion capacity of EVs while altering the RNA profile of pituitary cell-derived EVs (75). Insulin resistance can stimulate EV release and alter the levels of insulin signaling proteins in EVs (76). Cholesterol homeostasis plays a critical role in the uptake of extracellular vesicles, and lowering cholesterol levels in myeloid cells can inhibit receptor myeloid cell uptake of prostate cancer-derived EVs (77). Indeed, dysregulation in the number and composition of EVs is prevalent in various metabolic diseases (78). For example, significant changes occur in the number, location, and inflammatory status of ATMs during obesity. When incubated with EVs from human adipocyte lines and AT-derived EVs, monocytes will differentiate into ATMs-like macrophages, and conditioned media from these macrophages can significantly inhibit insulin signaling in adipocytes *in vitro* (51), further demonstrating that this is due to EVs released from M1 macrophages (69). Interestingly, EVs-like carriers from M1 macrophages impair insulin signaling in human adipocytes, while EVs-like carriers from M2 macrophages have the opposite effect (69). Similarly, EVs obtained from ATMs of lean mice, when given to obese mice, can improve glucose tolerance and insulin sensitivity, while injections of exosomes isolated from ATMs of obese mice induce glucose intolerance and insulin resistance in lean mice (8). Although the specific molecular mechanisms driving these changes remain unclear, they also indicate that the components contained in EVs released by specific cells change under metabolic disorder conditions, further causing metabolic dysregulation. In summary, ATMs-EVs can mediate crosstalk between key metabolic tissues and adipocytes and participate in the body’s metabolic regulation under physiological and pathological conditions.

**Figure 1:**
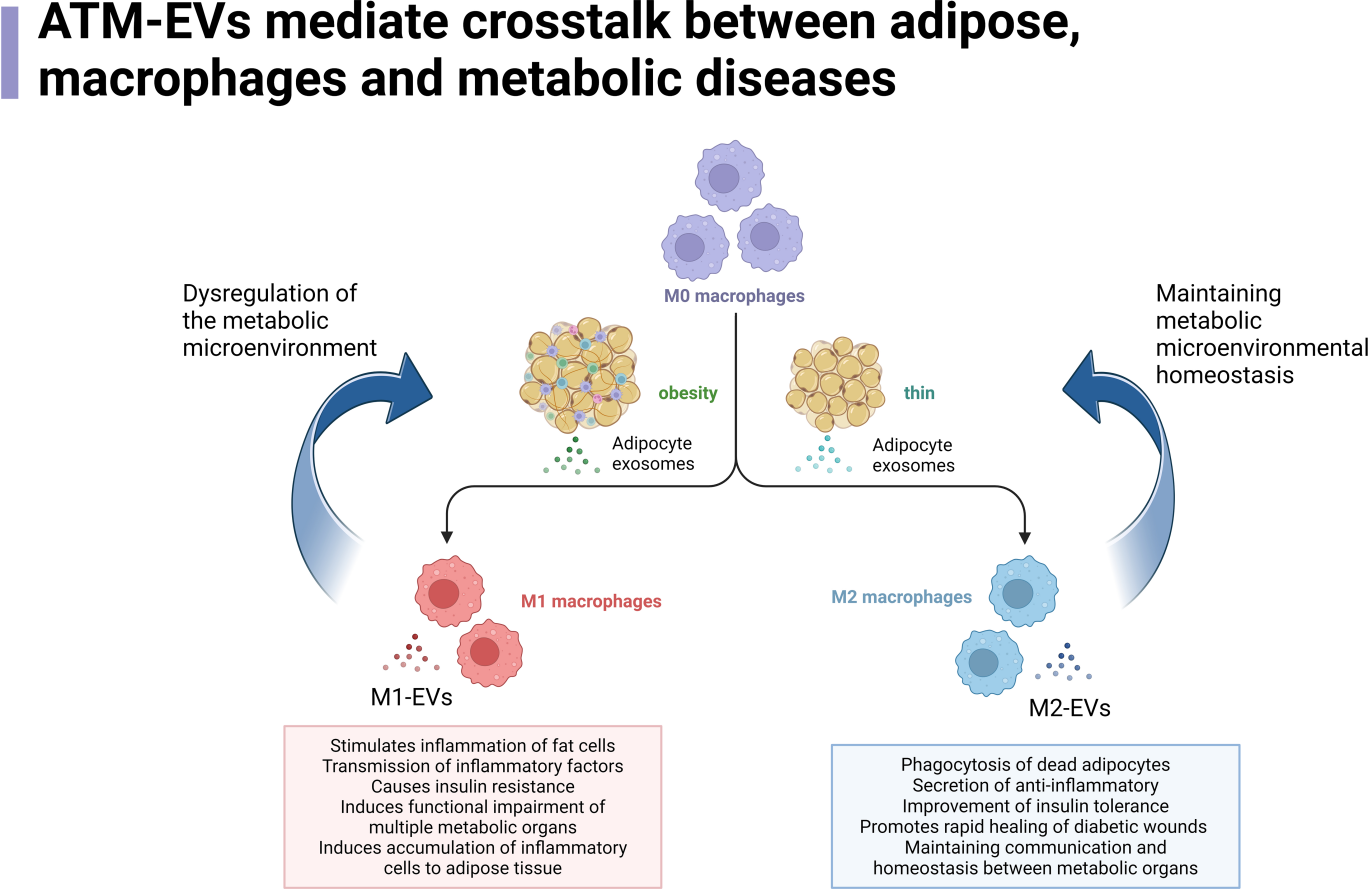
ATM-EVs mediate crosstalk between adipose, macrophages and metabolic diseases.

Interestingly, EVs seem to have a regulatory effect on the crosstalk between adipocytes and ATMs and insulin resistance (79). Research has found that ATMs-EVs miR-29a can participate in obesity-induced insulin resistance by targeting PPAR-δ (80). miR-29a belongs to the miR-29 family, which is widely present in EVs. miR-29 family proteins delivered by EVs can affect insulin resistance in obesity and diabetic patients by mediating glucose and lipid metabolism in adipocytes, myocytes, and hepatocytes (81). Interestingly, the level of miR-29a in urinary EVs is independently associated with obesity, insulin resistance, lipids, and liver enzymes, making it a potential biomarker for T2DM (80, 82). While EVs secreted by adipocytes have profound effects on ATMs polarization and function, ATMs themselves also produce EVs to regulate the metabolism and insulin action of local adipocytes and distant metabolic organs (55). Adipocyte-derived EVs increase due to obesity and indirectly assist ATMs in lipid uptake (83). In humans and mice, AT-derived EVs are a major source of circulating miRNA, and the miRNA content of adipocyte EVs in obese individuals differs significantly from that of lean individuals (23). Furthermore, changes in EVs miRNA are closely related to the reduction of insulin resistance after weight loss surgery. EVs miRNAs are key participants in mediating adipocyte-macrophage crosstalk within AT under physiological and pathophysiological conditions (84). In summary, ATMs-EVs play a crucial role in coordinating communication between adipocytes and other types of cells within AT, as well as between AT and other key metabolic organs (such as the liver and skeletal muscle) (85) **Figure 1**.

ATM-EVs mediate crosstalk between adipose tissue, macrophages, and metabolic diseases. In the context of obesity, M0 macrophages in adipose tissue undergo a transformation into M2 macrophages upon stimulation by EVs released from adipocytes. The M2 macrophages then regulate various pathways, such as promoting immune responses, transferring immune factors, altering the function of metabolic organs, and inducing immune cell aggregation, thereby creating a disrupted metabolic microenvironment that further promotes the continuous generation of M1 macrophages. In normal or lean conditions, M0 macrophages in adipose tissue are stimulated by EVs released from adipocytes to differentiate into M1 macrophages. These M1 macrophages, in turn, regulate processes such as promoting the breakdown of dead adipocytes, reducing inflammation, improving insulin sensitivity in metabolic organs, enhancing wound healing, and shortening the duration of inflammatory states. These processes help maintain the communication and stability of metabolic organs, sustaining a normal metabolic microenvironment while further promoting the generation of M2 macrophages.

## Possibility of ATMs-EVs modulating immunotherapy for metabolic diseases

The pathogenesis of metabolic diseases may be related to the activation or suppression of immune cells, which is based on altered communication between different organs. For example, the communication between the liver, pancreas, AT, and immune system may be associated with the convergence of immune cells or the potential transmission of information to activate immune cells within the tissue. In fact, changes in the metabolic microenvironment are significantly associated with the activation of immune cells. In the metabolic microenvironment experienced by immune cells within tumor tissues, nutrients can alter metabolic programming and form an anti-tumor immune response (86). The activity of immune cells is also affected by the availability of nutrients; under starvation conditions induced by infection, ketone bodies can directly affect the survival of CD4+ T cells and regulate their production of IFNγ (87). Conversely, immune cell dysfunction can be observed in states of nutrient excess, such as hyperglycemia and hyperlipidemia (88). These phenomena can be significantly manifested in the re-regulation of glucose under conditions of marked inflammation, such as sepsis and critical illness. Interestingly, the responsiveness of immune cells to changes in the nutrient state of the metabolic microenvironment is particularly evident in lipid-associated macrophages. This may help explain the development of various chronic diseases, such as heart-related metabolic diseases, caused by metabolic disorders. The specific intercellular communication between tissue-resident immune cells and metabolic cells has also been confirmed in fasting and refeeding experiments *in vivo* (89). In this context, there is a high level of interaction between the immune system and the metabolic microenvironment. As the vanguard of the immune environment, macrophages naturally become key participants in the development and progression of metabolic diseases, such as T2DM (90).

AT is a unique tissue that has a powerful impact on immune cell function. The field of AT immunobiology reveals how AT shapes immune cell function under conditions of metabolic stress, such as obesity. Evidence suggests that many metabolic and tissue-specific complications of obesity are associated with the activation of inflammatory cells and the loss of tissue homeostasis. Research focusing on intracellular metabolic pathways has found that AT can control the activation of immune cells and regulate their function, ultimately affecting the growth of host cells (91). Due to the contribution of AT macrophages in lean and obese states, they have been extensively studied. Macrophages are the most abundant immune cell population in obese AT, accounting for 40-60% of AT immune cells in obese mouse models (6). In obesity, the pro-inflammatory activity of ATMs can stimulate adipocytes to secrete pro-inflammatory mediators, such as TNF-α and IL-6, which in turn activate and recruit other immune cells (92). As the predominant immune cell in AT in terms of function and quantity, ATMs can regulate obesity-induced insulin resistance by altering the secretion of inflammatory and anti-inflammatory factors (55). The number of ATMs in obese mice and humans is significantly increased and positively correlated with obesity (6). Specifically, in obese mice, the number of activated M1 ATMs (typical inflammatory macrophages) increases, leading to an increased M1/M2 macrophage ratio (80). Furthermore, the accumulation of immune cells, including macrophages, produces a chronic inflammatory state associated with insulin resistance. AT macrophages have characteristics related to AT metabolic function, which differ from the typical characteristics of macrophages in other tissues. Studies have shown that various epigenetic changes caused by a hyperglycemic environment led to elevated inflammatory cytokine expression, promoting M1 macrophage polarization. The accumulation of M1 macrophages leads to chronic inflammation of AT and ultimately causes insulin resistance (93). Interestingly, some data suggest that ATMs have beneficial effects, such as increasing fat storage, regulating angiogenesis, remodeling the extracellular matrix, and clearing dead cells in AT to maintain AT homeostasis. Therefore, macrophages may have various or even opposite effects on adipocytes depending on the physiological conditions, which likely depends on the body’s metabolic state (52). For example, adipocyte-secreted microRNA-34a (miR-34a) can act as a key mediator through its paracrine effect on ATMs. Adipose-selective or adipocyte-specific resistance to obesity-induced glucose intolerance, insulin resistance, and systemic inflammation transmits the signal of nutrient excess to ATMs, thereby exacerbating systemic inflammation and metabolic dysregulation caused by obesity (84). In addition, AT hypoxia is a tissue-specific phenomenon that occurs during the rapid expansion of AT in obese individuals. ATMs isolated from obese AT exhibit a sustained elevated hypoxic state, implying that the pathophysiological role of ATMs is regulated by certain inflammation-related transcription factors induced by a combination of hypoxia and metabolic stress (94, 95). Most studies on AT HIF have focused on HIF-1α (96, 97). For example, macrophage HIF-2α can attenuate pro-inflammatory properties by inducing ARG1, thereby preventing pro-inflammatory responses and insulin resistance in adipocytes. In this regard, maintaining appropriate activity of HIF-2α is crucial for preventing AT dysfunction in obesity, suggesting that enhancing HIF-2α activity in ATMs may be an attractive approach for treating metabolic disorders caused by obesity (98). Persistent and unresolved inflammation and hypoxia in AT are major causes of obesity-related metabolic complications. However, the molecular link between lipid-overloaded adipocytes and inflammatory immune cells in obese AT remains elusive.

Interestingly, studies on AT-released EVs in obese individuals may explain this molecular link between the two cell types. For example, macrophage EVs in AT can induce the convergence of surrounding immune cells to AT, leading to the occurrence of metabolic complications. Studies have found that ATMs-EVs play an important role in immune surveillance, signal mediation, and promoting disease progression in the pathogenesis and pathology of inflammation and related diseases. ATMs-EVs can influence the chemotactic properties of peripheral immune cells by affecting the release of pro-inflammatory enzymes and cytokines, indicating that ATMs-EVs have pro-inflammatory or anti-inflammatory properties (58). ATMs-EVs produced in the AT of lean mice can directly reduce systemic immune responses *in vivo*, thereby promoting insulin signaling. When administered to obese mice, they can significantly improve insulin sensitivity and glucose tolerance (99, 100). Conversely, in type 2 diabetes, ATMs-EVs can mediate immune cell activation and insulin resistance (101), possibly due to the effects of EVs derived from inflammatory M1 macrophages on adipocyte differentiation and insulin signaling through NF-κB activation, while EVs derived from M2 macrophages enhance glucose uptake in adipocytes (69). In fact, once released into the extracellular space, ATMs-EVs regulate the metabolism of nearby and distant cells through body fluid circulation (92), although the specific process remains unclear. Additionally, it would be interesting to study whether the dysregulated EV miRNAs in macrophage EVs change after dietary or exercise interventions and understand the effects of ATMs-EVs on the systemic inflammatory response to further regulate the metabolic microenvironment (92). Although extensive studies have been conducted on the effects of macrophage EVs on the systemic immune response in the development of metabolic diseases and the impact of metabolic stress on the production of EVs by macrophages, the role of ATMs-EVs in metabolic pathology and whether they can prevent or even intervene in the occurrence or progression of metabolic diseases by acquiring EVs released by different types of macrophages to influence their immune responses, and constructing specifically needed ATMs-EVs to treat complications caused by metabolic diseases, still require sufficient research to be confirmed.

## Exploration of the clinical use of ATMs-EVs

**Figure 2:**
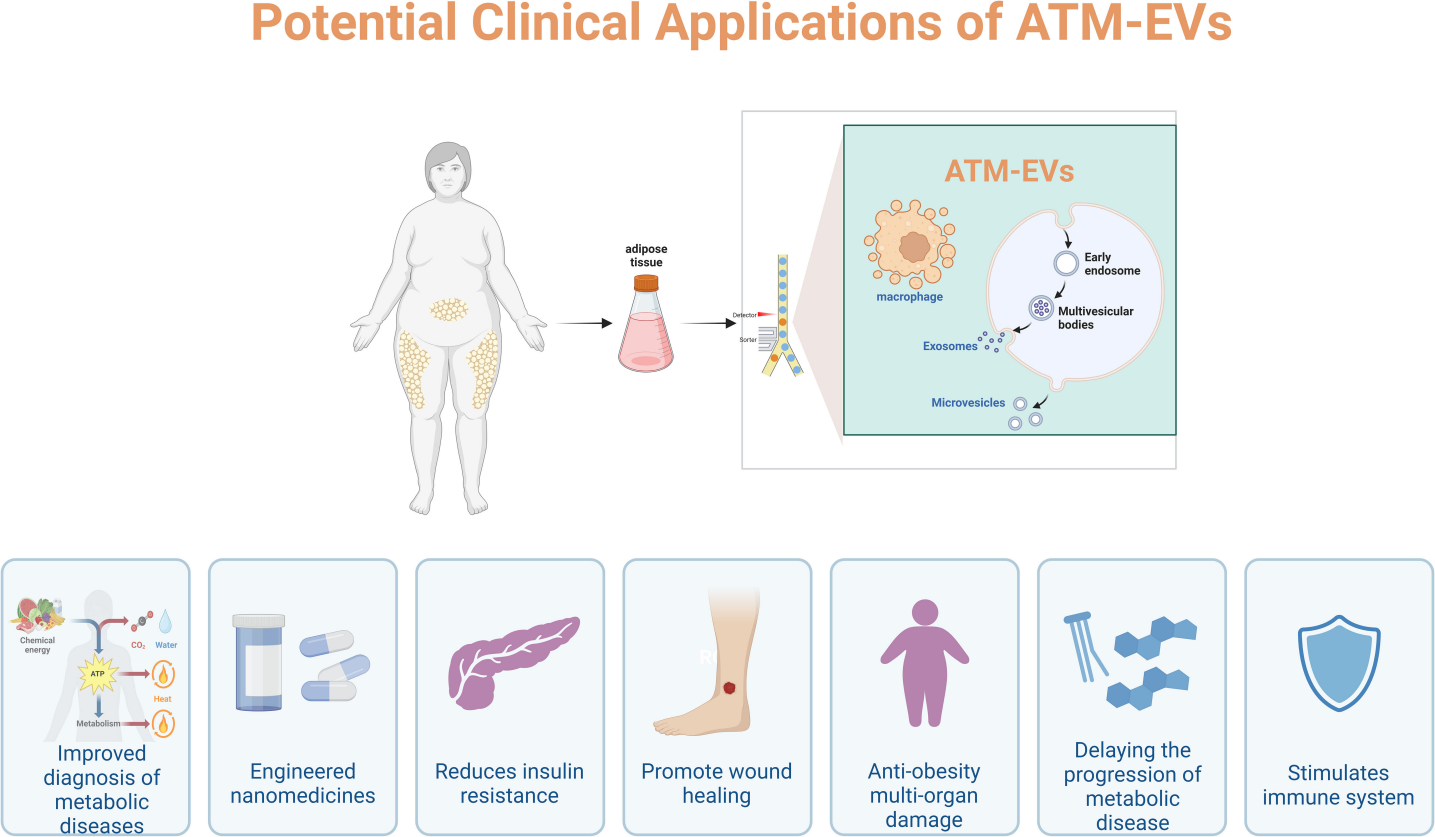
Potential clinical applications of ATM-EVs.

Today, approximately 500 million people worldwide are affected by metabolic disorders and their complications. The World Health Organization (WHO) estimates that this number will increase to around 700 million by 2045 due to unhealthy lifestyles (92). Currently, significant efforts are being made to prevent and treat metabolic complications, and new discoveries in the field of EVs have encouraged researchers to consider these naturally constructed nanovesicles for clinical applications. Due to their potential to manipulate drug delivery, specific targeting, and homing properties, EVs are considered “professional transporters and messengers” at the systemic level in the body (102–104). Because exosomes protect their cargo from degradation by circulating enzymes, all these characteristics provide potential for disease diagnosis and evaluation of the efficacy of specific drugs. In fact, EVs are considered very attractive nanocarriers or biomarkers for liquid biopsy due to the protection of their molecular cargo by a lipid bilayer membrane (105). This makes EVs particularly suitable as a source of liquid biopsy for various diseases, including post-obesity metabolic disorders and inflammatory responses (106, 107). As the global obesity epidemic becomes a major driving force behind the increasing prevalence of T2DM, obtaining a method to enhance insulin sensitivity would have significant clinical application value. Studies have found that treatment with obese ATMs-EVs leads to reduced insulin secretion and enhanced β-cell proliferation both *in vivo* and *in vitro* (31). It was observed that insulin signaling in cells improved significantly after *in vitro* or *in vivo* experiments using M2-like macrophage EVs highly enriched in miR-690. New evidence suggests that insulin-sensitive lean mice secrete EVs containing miRNA from ATMs, which can be transported to insulin target cells to promote insulin sensitivity (108). Conversely, M2-EVs treatment enhances insulin sensitivity both *in vivo* and *in vitro*, while inhibiting M2-EVs miRNA can prevent these effects (108). Interestingly, the secretion levels of ATMs-EVs have distinctly different impacts on various diseases, reflecting the influence of different cell types and environments on the control of EVs secretion. In fact, ATMs-EVs play key roles in treating diseases such as cancer, atherosclerosis, diabetes, heart disease, and inflammation (109–113). It has been reported that all types of M2-EVs can alleviate the severity of inflammatory bowel disease, with M2b-EVs (M2b macrophage-derived EVs) having the best effect. Additionally, ATMs-EVs can be used as tools for drug delivery, or as vectors for gene or protein delivery (114) **Figure 2**. In terms of metabolic diseases, it has been found that M1-EVs help reduce inflammation in AT and insulin resistance of cells. For example, miR-27-3p in M1-EVs can regulate inflammation and insulin resistance mediated by mitochondrial autophagy defects through the miR-27-3p-Miro1 axis and has been confirmed to have beneficial effects in preventing the development of type 2 diabetes. This could provide new therapeutic targets for T2DM (115). Moreover, by purposefully engineering ATMs-EVs, they could become ideal functional carriers for delivering genetic material and drugs to specific disease sites for targeted treatment. For example, the engineered ATM2-EVs@PMN, through the combined effect of M2-EVs and PMN-generated photothermal effects, can inhibit inflammation and drive angiogenesis to promote diabetic wound healing, which could be a promising cell-free approach to treating metabolic diseases (58).

In terms of systemic metabolism, intercellular communication is crucial for coordinating the activities of important organs such as the brain, pancreas, liver, muscle, and AT. It is generally believed that non-synaptic intercellular communication occurs either locally through paracrine signaling or over longer distances through endocrine signaling (body fluids), both involving the secretion of signaling molecules such as growth factors, cytokines, and hormones. However, intercellular communication via the secretion of EVs has recently been considered an important driver of intercellular/inter-organ signal transduction, as EVs allow vesicles carrying specific molecular information to target and deliver it to specific cells anywhere in the body in a timely manner. This helps change the traditional view of intercellular communication and represents an alternative and universal mode of intercellular communication based on molecular cargo (i.e., proteins, lipids, nucleic acids, and membrane receptors) (92). This type of intercellular communication induces a wide range of stimulating or inhibitory functional outcomes, including cell proliferation, apoptosis, cytokine production, immune regulation, and metastasis (116). Therefore, they add an alternative mode of paracrine and endocrine communication beyond the traditional strategies of cell-to-cell direct contact and soluble receptor-targeting hormones and cytokines. The selective delivery of signaling molecules by EVs may be one of the reasons for the complexity of diseases. In obese rodents and humans, the protein (including adipokines) and RNA content of ATMs-EVs show qualitative differences (117). A study of the ATMs-EVs miRNA profile in 219 patients observed different ATMs-EVs miRNA profiles in metabolic syndrome, T2DM, hypercholesterolemia, and hypertension (118). These ATMs-EVs can interact with recipient cells, delivering their cargo into the cytoplasm of recipient cells and regulating their phenotype. ATMs-EVs can deliver not only functional proteins and translatable miRNA (119, 120) but also their miRNA cargo can silence target genes in recipient cells (121). Thus, we can achieve cell-level therapy by delivering the desired cargo to target cells through ATMs-EVs internalization or by targeting the action of ATMs-EVs surface molecules on target cells (122). Due to the lack of sufficient immunogenicity, EVs can be engineered for the clinical treatment of metabolic diseases (123). EVs are highly complex vesicles whose bilayer structure and cargo transfer capabilities allow them to serve as natural carriers for therapeutic drugs and prevent their degradation in the body. Currently, EVs loading technology can be divided into endogenous loading (genetic modification of parent cells) and exogenous loading (drug loading of EVs) (58), of course, this is not related to the specificity of ATMs-EVs. Therefore, to better utilize the special effects of ATMs-EVs (originating from obese or healthy individuals) and avoid biohazards and reduced metabolic regulation caused by human intervention, directly using ATMs-EVs from original tissue sources may have greater clinical value. However, for practical application, determining the optimal injection dose, timing, route, and rate of ATMs-EVs is important for enhancing clinical efficacy and reducing side effects.

## Discussion and prospects

Before ATMs-EV-mediated cell communication can be used for therapeutic purposes, more research is needed. Little is known about the recruitment and packaging of exosome cargo and the processes involved in targeting exosomes to specific target cells. Questions remain about whether EVs cargo loading and targeting addresses vary with different metabolic states, how these processes are regulated, and the characteristics of ATMs-EVs induced between different metabolic cell types and metabolic organs (79). The differences in protein and RNA content within ATMs-EVs increase the possibility that they may act in a nonlinear fashion at multiple stages of a single signaling pathway in metabolic processes or operate on multiple pathways. For instance, they can also indirectly act by stimulating metabolic cells to release signaling peptides or receptor ligands, or by mediating the intracellular transfer of lipid-insoluble signaling molecules (124, 125). These choices largely depend on the material composition of individual exosomes and are related to insulin resistance and impaired insulin signaling (91). Moreover, there are many differentially expressed macrophage-derived exosomal miRNAs between lean and obese states, with relatively highly expressed miRNAs thought to have biological effects. These studies not only emphasize the importance of macrophages as a source of adipose exosomes but also indicate that ATMs can produce EVs containing different types of cargo depending on their phenotype. Since various macrophage populations coexist within AT (36), characterizing the types of ATMs-EVs produced by these different populations in health and metabolic disease is crucial to understanding the specific roles of inflammatory cells (126). Although a series of mammalian cells have been used to study ATMs-EVs subpopulations, there is a lack of systematic deep characterization of all EVs populations within single-cell types from multiple sources (i.e., resident macrophages from different organs or tissues). Identifying differences between the same ATMs-EVs in different metabolic microenvironments may help better distinguish pathological EVs signals of metabolic disease phenotypes in peripheral blood. Currently, research on metabolic ATMs-EVs primarily focuses on EVs miRNA or EVs proteomics, with limited efforts directed towards exploring the synergistic or coordinated disease characteristics within the EVs RNA-proteome combination. This combined approach could support the possibility that ATMs-EVs are produced under specific disease conditions and promote the progression of metabolic diseases through direct cellular targeting (126). Furthermore, multiple miRNAs within ATMs-EVs might act in a coordinated manner to induce insulin resistance and insulin-sensitive phenotypes. It might be crucial to demonstrate the full spectrum of metabolic effects induced by ATMs-EVs miRNAs. Regarding systemic metabolic regulation, it is important to determine whether macrophages in the liver, AT, and skeletal muscle express the same miRNAs within EVs, and whether ATMs-EVs and their associated miRNAs specifically circulate to the liver and skeletal muscle to alter tissue-specific or systemic metabolic responses. Further clarification of the process by which macrophage recruitment is regulated and the phenotypic changes of ATMs could potentially decipher the methods for using ATMs-EVs to treat obesity and suppress chronic metabolic diseases caused by inflammation in systemic AT (127). Under conditions of metabolic disorders, immune cells that play a key role in nutritional regulation appear to be activated in all tissues, leading to sustained damage to the homeostatic functions of the cardiovascular system, brain, pancreas, liver, and AT. ATMs-EVs, as one of the key mediators, might contribute to the damage to systemic metabolic cells due to the collective effect of all ATMs-EVs. Identifying the predominant type of ATMs-EVs and their cargo in different metabolic diseases could facilitate early intervention in the onset of chronic metabolic diseases.

In fact, further research is needed on the functional changes in M1 and M2 polarization within obese AT and their respective EVs. Given that the recruitment and polarization of ATMs is a complex process regulated by various metabolic and immune factors, it remains to be determined how multiple regulatory factors communicate and coordinate to control the number and characteristics of ATMs during the development of obesity (84). ATMs-EVs play a role in controlling inflammatory responses in various metabolic diseases, including hypertension and diabetes. However, the specific targets and roles of ATMs-EVs in regulating disease-related inflammation are largely unknown. Although a significant number of miRNAs associated with the pathogenesis of T2DM have been identified in exosomes derived from AT macrophages, their pathogenic roles remain unclear. Specifically, further studies are needed to investigate the role of M1-EVs in human islets and assess whether targeting miRNAs or inhibiting M1-EVs could mitigate β-cell damage in rodent models and patients with type 2 diabetes (128). However, due to the low number of ATMs in lean and healthy mice, it is challenging to harvest enough macrophages from lean AT for more in-depth mechanistic studies.

Before the clinical application of ATMs-EVs, some fundamental issues still need to be addressed, such as how EVs target specific cells *in vivo*, whether there are specific markers to identify different organs, whether the same EVs cargo exerts similar functions in different target organs, and the safety, dosage, and bioavailability of EVs for treating metabolic diseases *in vivo*. Clarifying these issues and further modifying or interfering with the communication of these EVs cargos may provide potential therapeutic strategies for treating metabolic diseases.
